# Traumatic neuroma in a patient with breast cancer after mastectomy: a case report and review of the literature

**DOI:** 10.1186/1477-7819-10-35

**Published:** 2012-02-13

**Authors:** Quan Li, Er-li Gao, Yin-long Yang, Hong-ye Hu, Xiao-qu Hu

**Affiliations:** 1 Department of Surgical Oncology, The First Affiliated Hospital of Wenzhou Medical College, Wenzhou, Zhejiang, People's Republic of China; 2 Department of Surgical Oncology, Wenzhou Medical College, Wenzhou, Zhejiang, People's Republic of China

**Keywords:** Traumatic neuroma, Breast neoplasms, Mastectomy

## Abstract

The incidence of traumatic neuroma is extremely low, especially in those patients with breast cancer after mastectomy. There are only 10 cases reported in the literature. We report a patient who developed a palpable nodular mass near the mastectomy scar. The result of excisional biopsy was traumatic neuroma. Review of the literature reveal 10 cases with breast cancer of traumatic neuromas after mastectomy. Traumatic neuroma is a benign lesion and a reparative response of the nerve to injury, either direct/indirect trauma or chronic inflammation. Benign lesions as traumatic neuromas are more rarely seen after mastectomy. However, in order to manage patients' treatment, the most critical problem is to distinguish it from recurrent breast carcinoma. Although assistant examination methods such as ultrasound and computed tomography are valuable to a certain extent, the final diagnosis can only be confirmed on pathologic examination.

## Background

Traumatic neuromas are uncommon benign lesions, especially being the consequence of surgical procedures [1,2]. A traumatic neuroma is not a true neoplasm. It represents a hyperplastic, reparative response of the nerve to injury and usually presents as a palpable nodule mass. It is secondary to the disordered proliferation of nerves and connective tissue trying to renervate the area after a total or partial sectioning of anerve during an accidental or surgical trauma [3]. Traumatic neuromas occurring near the mastectomy scar are more rare. The first reported case was in 2000 and there have been only a total of 10 published cases [4-8]. Although post-mastectomy traumatic neuromas are benign lesions, they have to be distinguished from recurrent breast carcinomas to better manage patients' next treatment.

We present herein a further case of post-mastectomy traumatic neuroma and review the literature on the rare condition.

## Case report

A 43-year-old yellow female noticed a lump in the right breast in 2007. Examination at that time revealed a palpable mass without pain and the size of it was about 3 × 2 cm.

Ultrasonographic examination showed an irregular, solid, hypoechoic mass in the right breast. The internal echo texture was considered heterogeneous. There was no abnormality in her left breast. This finding can permit us to suspect that the mass is malignant. The next day, the patient underwent right-sided modified radical mastectomy (MRM). Histopathological examination showed T2N0M0 invasive ductal carcinoma of the breast. One week after the surgery, the patient started to receive six cycles of taxol-based chemotherapy. In consideration of her ER and PR status, the patient was treated with tamoxifen (TAM).

**Figure 1:**
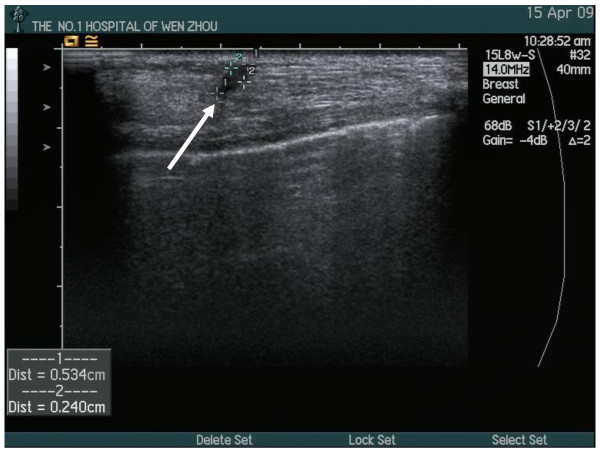
**Ultrasound examination shows a well-circumscribed, echo-heterogeneous, subcutaneous nodular lesion, with a max diameter isφ0.5 cm**.

**Figure 2:**
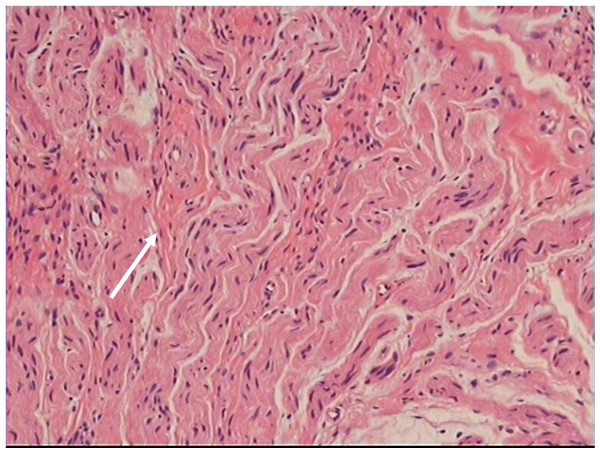
**A tangle of small and medium nerve fibre bundles which were well circumscribed in fibro-adipose tissue**.

A nodular mass was detected at the chest wall 2 years after the surgery. The nodular mass was close to the mastectomy scar and was palpated at follow-up examination. Ultrasonographic examination showed a well-circumscribed, echo-heterogeneous, subcutaneous nodular lesion, with a diameter of about 0.5 cm. (Figure 1) There was no obvious indicator to identify the distant metastases. In order to confirm whether the mass was a recurrence cancer or not, an excision biopsy was performed of the presenting mass. Histopathological examination showed a tangle of small and medium nerve fiber bundles which were well circumscribed in fibro-adipose tissue. (Figure 2) Hence, we could confirm that the nodular mass was traumatic neuroma and there was no recurrence.

## Discussion

A review of the literature searching for patients with breast cancer and traumatic neuroma revealed a total of 10 reported cases [4-8]. This article presents an additional case with breast cancer of traumatic neuroma occurring in a patient 2 years after mastectomy and reviews the previously reported cases.

Table 1 summarizes the clinical circumstances for the 10 reported cases plus this report of traumatic neuroma after mastectomy for breast cancer. The age of the patients at presentation was variable, ranging from 41 to 68 years. The time between mastectomy and presentation with traumatic neuroma was also variable in these 11 cases (2 to 14 years after mastectomy) and the average was 5.3 years. There was only one patient who had three nodules near the mastectomy scar and all other patients had only one nodule. The diameter of every nodule ranged from 0.4 to 1.0 cm. There were 7 patients whose traumatic neuromas were located near the scar of mastectomy, 2 patients whose traumatic neuromas were located in the subclavian area, one patient whose traumatic neuroma was in the axilla and one patient whose traumatic neuroma was located in the 9 o'clock position in the right chest wall. Eight patients underwent ultrasound examination. Reports showed five cases with well-circumscribed, homogeneous, hypo-echoic subcutaneous nodular lesion and two cases with a poorly defined hypo-echoic nodule with good conduction. The latest report from Kim EY et al showes that it was an oval, circumscribed, hypo-echoic nodule with a partially microlobulated margin [8]. Two cases in one report didn't mention whether they underwent ultrasound examination or not. Ultrasound examination of the case we present showed that the mass was a well-circumscribed, echo-heterogeneous, subcutaneous nodular lesion, with a diameter of about 0.5 cm.

**Table 1:** Clinical datas for reported cases of traumatic neuroma after mastectomy for breast cancer

Source	Age(yr)	Period of neuroma after mastectomy(yr)	The amount and diameter of nodules	Location	Ultrasound description	Pain
Present case	45	2	One; φ = 0.5	Close to the mastectomy scar	Well circumscribed, echo-heterogeneous, subcutaneous nodular lesion	-
Xing Wang et al,	68	7.5	One; φ = 0.7 cm	Close to the mastectomy scar	Well circumscribed, homogeneous, hypo-echoic	-
2007[5]					subcutaneous nodular lesion	
Xing Wang et al,	45	2.83	Three;φ = 0.8 cm, 0.6 cm, 0.4 cm	Close to the mastectomy scar	Three well defined, homogeneous,	-
2007[5]					hypo-echoic nodules	
Xing Wang et al,	57	8	One; φ = 0.7 cm	Subclavicular	Poorly defined hypo-echoic nodule with	-
2007[5]					good conduction	
Xing Wang et al,	43	4	One; φ = 1.0 cm	Axilla	Poorly defined hypo-echoic nodule with	-
2007[5]					good conduction	
Xing Wang et al,	41	8	One; φ = 1.0 cm	Subclavicular	Homogeneous, hypo-echoic subcutaneous	-
2007[5]					nodular lesion	
Xing Wang et al,	52	3	One; φ = 0.9 cm	Close to the mastectomy scar	Well defined, homogeneous, hypo-echoic	-
2007[5]					subcutaneous nodular lesion	
Rosso R et al,	55	2.33	One; φ = 0.4 cm	Close to the mastectomy scar	Not mentioned	-
2000[4]						
Rosso R et al,	67	4.17	One; φ = 0.6 cm	Close to the mastectomy scar	Not mentioned	-
2000[4]						
B Baltalarli et al	54	2	One; φ = 1.0 cm	On the mastectomy scar	Well circumscribed, homogeneous, hypo-	-
2004[7] Kim EY et al, 2011[8]	47	14	One; φ = 1.0 cm	In the 9 o'clock position in the right chest wall	echoic subcutaneous nodular lesions Oval, circumscribed, hypo-echoic nodule with a partially microlobulated margin	-

φ = diameter -: Negative

All of the nodules were painless. All of these cases received excision biopsy of the presenting nodules and made a definite diagnosis by histopathological examination.

Table 2 summarizes the diagnosis and treatment of breast cancer for reported cases. The age at diagnosis of breast cancer was variable, ranging from 33 to 63 years. Four patients' stage was T1N0M0, five patients' stage was T2N0M0, and two patients' stage was T2N1M0. Pathological examination of all the patients was invasive ductal carcinoma of the breast. A majority of patients underwent a radical or modified radical mastectomy (There were two cases of a report did not mention the kind of surgery). Seven patients received chemotherapy (specific chemotherapy regimen and cycles are recorded in the Table). Two patients received radiotherapy and five patients received endocrinotherapy in consideration of their ER and PR status (four patients treated with tamoxifen and one patient treated with aromatase inhibitor).

**Table 2:** The diagnosis and treatment of breast cancer for reported cases

Source	Age at diagnosis(yr)	Tumor size (cm)	Stage	Kind of surgery	Lymph nodes metastasis	Chemotherapy	Radiotherapy	Endocrinotherapy	ER	PR
Present	43	3 × 2	T2N0M0	MRM	0/11	TEC, 6 cycles	-	TAM	+	+
case										
Xing Wang et al,	61	2 × 2	T1N0M0	RM	0/19	CU, 4 cycles	-	TAM	+	+
2007[5]										
Xing Wang et al,	42	3.5 × 3.5	T2N0M0	MRM	0/24	CMF, 6 cycles	Only internal	-	-	+
2007[5]							Mammary area			
Xing Wang et al,	49	4 × 4	T2N1M0	MRM	4/20	CEF, 6 cycles	-	TAM	+	-
2007[5]										
Xing Wang et al,	39	2.5 × 2.5	T2N0M0	MRM	0/18	CEF, 4 cycles	-	TAM	+	+
2007[5]										
Xing Wang et al,	33	1.5 × 1.5	T1N0M0	MRM	0/15	-	-	-	-	-
2007[5]										
Xing Wang et al,	49	5 × 4	T2N1M0	MRM	3/10	CEF, 6 cycles	-	TAM	-	+
2007[5]										
Rosso R et al,	53	/	T1N0M0	MRM	/	-	-	/	/	/
2000[4]										
Rosso R et al,	63	/	T1N0M0	/	/	-	-	/	/	/
2000[4]										
B Baltalarli et al,	52	/	T2N0M0	/	/	Anthracycline-based	External	AI	/	/
2004[7] Kim EY et al, 2011[8]	33	/	T2N0M0	MRM	/	chemotherapy, 4 cycles Adjuvant chemotherapy for 1 year*	Radiotherapy -	/	/	/

/: Not mentioned in the report +: Positive -: Negative or without treatment RM: Radical mastectomy. MRM: Modified Radical Mastectomy TEC: Taxol/Epirubicin/Cyclophosphamide CMF: Cyclophosphamide/Methotrexate/Fluorouracil CU: Colchicines/Uraphetine CEF: Cyclophosphamide/Epirubicin/Flurouracil TAM: Tamoxifen treatment AI: Aromatase inhibitor treatment *: No mention of specific d

Neuromas occurred in patients who have received cancer surgery are not well established in the literature, unlike traumatic neuromas following amputation for benign diseases such as post-cholecystectomy [2]. A traumatic neuroma is not a true neoplasm. It represents a hyperplastic reponse of the nerve to injury, either direct/indirect trauma or chronic inflammation. Traumatic neuromas show disrupted axons with distal demyelination and Wallerian degeneration, and a tangled mass of Schwannou cells and fibroblasts, all in a dense, collagenous matrix [3]. Enzinger FM et al [9] and Murphey M at el [10] consider that a traumatic neuroma usually presents as a palpable nodule which is caused by a non-neoplastic proliferation of the proximal end of a severed, partially transected or injoured nerve after surgery and it dosen't have any potential malignancy. Although most traumatic neuromas affect peripheral sensory nerves, they could also affect motor sensory nerves and autonomic nervous system [11]. The most frequent site of traumatic neuroma is at radically dissected neck, followed by upper and lower extremities [12].

Benign lesions as traumatic neuromas after mastectomy are more rarely seen. The first reported case was in 2000 and there have been only a total of ten published cases. Two of them resembled granular cell tumours at mastectomy. Sometimes there was pain in tumor sites but all of the post-mastectomy patients in the review were asymptomatic. Hence, a traumatic neuroma after mastectomy is an uncommon entity, but it should be kept in mind because the most critical problem is to distinguish the painless nodule from recurrent breast carcinoma. Assistant examination methods such as ultrasound and computed tomography are valuable to a certain extent. Kim EY et al [8] suggested that if a color Doppler image shows no increased blood flow, fluorodeoxyglucose (FDG) positron emission tomography-computed tomography (PET-CT) shows no significant uptake, and the time interval between surgery and the development of new lesion is more than 5 years, one should include traumatic neuromas in the differential diagnosis. Huang LF et al [13] and Yabuuchi H et al [2] also suggested that several imaging techniques such as ultrasonographic, computed tomographic, and magnetic resonance imaging features have been used to differentiate traumatic neuroma from recurrent lymphadenopthy after neck dissection. Nevertheless, the final diagnosis can only be confirmed on pathologic examination.

## Conclusion

It is very difficult to diagnose traumatic neuromas occurring in mastectomy scars because they can mimic recurrent lymph nodes or local tumor recurrence. In order to manage patients' treatment, the most critical problem is to distinguish it from recurrent breast carcinoma. Although some assistant examination methods are valuable to a certain extent, the final diagnosis can only be confirmed on pathologic examination.

## Consent

Written informed consent was obtained from the patient for publication of this Case report and any accompanying images. A copy of the written consent is available for review by the Editor-in-Chief of this journal.

## List of abbreviations

MRM: Modified radical mastectomy; TAM: Tamoxifen; RM: Radical mastectomy; TEC: Taxol/Epirubicin/Cyclophosphamide; CMF: Cyclophosphamide/Methotrexate/Fluorouracil; CU: Colchicines/Uraphetine; CEF: Cyclophosphamide/Epirubicin/Flurouracil; AI: Aromatase inhibitor treatment; FDG: fluorodeoxyglucose; PET-CT: positron emission tomography-computed tomography.

## Competing interests

The authors declare that they have no competing interests.

## Authors' contributions

LQ carried out the initial conception and design as well as collection of data and clinical records of the patient. GEL participated in its design and helped to edit the manuscript. HXQ and LQ made up the surgical team involved in the case and carried out the initial conception. YYL and HHY help to revise the manuscript. All authors read and approved the final manuscript.

## Authors' information

Li Q is a surgeon of department of Oncology, The First Affiliated Hospital of Wenzhou Medical College, Wenzhou, Zhejiang, People's Republic of China

Yang YL is a surgeon of department of Oncology, The First Affiliated Hospital of Wenzhou Medical College, Wenzhou, Zhejiang, People's Republic of China

Gao EL is a graduate of Wenzhou Medical College, Wenzhou, Zhejiang, People's Republic of China

Hu HY is a graduate of Wenzhou Medical College, Wenzhou, Zhejiang, People's Republic of China

Hu XQ is associate Chief Physician of department of Oncology, The First Affiliated Hospital of Wenzhou Medical College, Wenzhou, Zhejiang, People's Republic of China
